# Defining a Regulatory Strategy for ATMP/Aerosol Delivery Device Combinations in the Treatment of Respiratory Disease

**DOI:** 10.3390/pharmaceutics12100922

**Published:** 2020-09-26

**Authors:** Niamh Woods, Ronan MacLoughlin

**Affiliations:** 1 College of Medicine, Nursing & Health Sciences, National University of Ireland, H91 TK33 Galway, Ireland; n.woods7@nuigalway.ie; 2 School of Pharmacy & Biomolecular Sciences, Royal College of Surgeons in Ireland, D02 YN77 Dublin, Ireland; 3 School of Pharmacy and Pharmaceutical Sciences, Trinity College, D02 PN40 Dublin, Ireland; 4 Aerogen Ltd., Galway Business Park, H91 HE94 Galway, Ireland

**Keywords:** ATMP, regulatory, aerosol, inhalation, nebulizer, extracellular vesicles, conditioned media, mesenchymal stromal cells, secretome, respiratory disease

## Abstract

Advanced Therapeutic Medicinal Products (ATMP) are a heterogenous group of investigational medicinal products at the forefront of innovative therapies with direct applicability in respiratory diseases. ATMPs include, but are not limited to, stem cells, their secretome, or extracellular vesicles, and each have shown some potential when delivered topically within the lung. This review focuses on that subset of ATMPs. One key mode of delivery that has enabling potential in ATMP validation is aerosol-mediated delivery. The selection of the most appropriate aerosol generator technology is influenced by several key factors, including formulation, patient type, patient intervention, and healthcare economics. The aerosol-mediated delivery of ATMPs has shown promise for the treatment of both chronic and acute respiratory disease in pre-clinical and clinical trials; however, in order for these ATMP device combinations to translate from the bench through to commercialization, they must meet the requirements set out by the various global regulatory bodies. In this review, we detail the potential for ATMP utility in the lungs and propose the nebulization of ATMPs as a viable route of administration in certain circumstances. Further, we provide insight to the current regulatory guidance for nascent ATMP device combination product development within the EU and US.

## 1. Introduction

Advancements in science and technology have seen the development of new therapeutic opportunities for the treatment and prevention of high-burden human diseases with unmet medical needs [1]. Advanced therapeutic medical products (ATMP) are at the forefront of innovation and are changing the therapeutic landscape for an array of human diseases [2]. ATMP is a broad term used to group three medicinal product classes intended for human use which encompass somatic cell therapy medicine, gene therapy medicines, and tissue-engineered medicines [3].

The three classes of ATMPs are highly heterogeneous in terms of their origin, type, and use [4]. The definition of each class is established in the relevant regulatory documents which define somatic cell therapy medicines (sCTMPs) as products which contain or are comprised of cells or tissues which have been modified for intended clinical use, or biological products which are transferred from one patient to donor with a different use [5]. Gene therapy medicine products (GTMPs) contain recombinant nucleic acids which have a therapeutic, prophylactic, or diagnostic effect relevant to the recombinant nucleic acid sequence [5]. Tissue-engineered products consist of engineered cells or tissues and present as having properties for or are used in or administered to humans with the view of regenerating, repairing, or replacing a human tissue. Some ATMPs may contain one or more medical device as an integral component of the medicine; this is defined as a combined ATMP (cATMP) [3,5]. This review will focus solely on somatic cell therapy medicine and its utility in respiratory disease, and details the current regulatory hurdles that need to be addressed in bringing a ATMP device combination product to market.

## 2. Cellular Therapy

Cellular therapy is a field of medicine which has rapidly progressed from research to clinical trials for many disease states [6]. Cellular therapy is based on the principles of rejuvenation, regeneration, and replacement and is converting the currently used symptomatic treatment to a personalized, curative treatment [7]. The development of cellular therapy over the past couple of decades has seen a variety of different therapies receive approval from regulatory bodies such as the Food and Drug Association (FDA) and the European Medicine Authority (EMA) and are available commercially on the market [8]. The cells used in these therapies are either autologous or allogenic, and although they are usually in a differentiated state the cells still retain their proliferative capacity [9]. For example, Kymriah^®^ is a recently approved FDA and EMA cellular product used in the field of immunotherapy which utilizes autologous T cells reprogrammed using a lentiviral vector to target diffuse large-blood-cell lymphoma. The cells are harvested from a patient, expanded, and modified ex vivo before being re-infused back to the same patient, where the results have been shown to be comparable to those of standard chemotherapy approaches [10].

### 2.1. Mesenchymal Stromal Cells

Cellular therapies such as stem, progenitor, and stem cell derivates show potential in the regulation of pathogenic mechanisms and the promotion of tissue repair and regeneration [11]. Owing to their “stemness” property, the cells hold great promise for the development of new therapeutic approaches for the treatment of respiratory diseases [12,13]. One cell type, in particular, under intense investigation for its potential utility in the treatment of a variety of human diseases are mesenchymal stromal cells (MSCs). The interest in MSC therapy is largely due to their accessibility from adult tissue, their multilineage differentiation potential, and their diverse mechanism of action through both cryoprotective and paracrine effects, as well as their low immunogenicity [14,15,16].

There have been many in vitro and preclinical studies demonstrating the potential use of MSCs in the treatment of different respiratory diseases, shown in their ability to reduce the inflammation associated with asthma patients and the regeneration of damaged tissue associated with COPD, for example [17,18]. Despite advancements in our understanding of MSCs and their therapeutic potential, until the results from the ongoing clinical trials using MSCs are released, determining the safety and efficacy of MSC administration for respiratory disease, widespread application cannot be considered.

### 2.2. The Secretome

The secretome is defined as the set of factors/molecules secreted to the extracellular space. These factors include, among others, soluble proteins, free nucleic acids, lipids, and extracellular vesicles [19]. The pro-regenerative effects and diverse mechanistic effects exerted by MSCs are achieved through the activity of the secretome [15]. Following the revelation that MSCs act through a paracrine mechanism, multipotency was no longer the main factor contributing to their therapeutic use; instead, MSCs were recognized as drug stores, reflected in their ability to secrete a plethora of biological materials [20]. 

The MSC secretome is made up of a cocktail of bioactive components such as anti-inflammatory factors, nucleic acids, cytokines, growth factors, and extracellular vesicles (EV), which play a significant role in the regulation of a range of physiological processes, thereby potentially making the MSC secretome a high potential target in exerting a therapeutic effect for the treatment of multiple respiratory diseases [21,22,23] The therapeutic effects include immunomodulation and anti-inflammatory activity, tissue repair, pro-angiogenic and antimicrobial activity, and the restoration of the protease/anti-protease activity associated with a variety of respiratory diseases [23]. 

The secretome is highly dynamic and adapts easily to culture conditions; the components may differ subject to donor characteristics or the microenvironment, and, owing to this, MSCs can be primed or preconditioned in order to condition or tailor the cells for maximum benefit [24,25]. MSCs potential in the treatment of any given disease may differ depending on their source, but this conditioning can be used to influence the phenotype, multilineage potential, and immunomodulating capacity of MSCs, ultimately impacting their therapeutic effect [26,27,28]. 

Although MSCs hold promise as a cell-based therapy option due to the wide range of therapeutic effects they exert, pre-clinical and clinical trials have highlighted some associated limitations, such as the high cell count doses required, complex storage requirements, and short shelf lives [19,29]. The MSC secretome offers a cell free therapy which can overcome cell-based therapy limitations [30]. Aside from preparing the cell to obtain a desired effect, the secretome overcomes safety considerations of MSC transplantation, such as tumorgenicity, and bypasses the need to deliver large amounts of cells. Moreover, the multi-component secretome can be stored after conditioning, potentially providing an off-the-shelf product readily accessible for the potential treatment of acute respiratory diseases and associated exacerbations, and can be stored without the use of cryoprotectants for a long period of time without compromising the safety of the product [19,25,31,32]. 

Owing to these factors, the secretome is at the forefront of research and development and is under investigation in a clinical setting in the form of conditioned media (CM) and EVs such as exosomes [15,33].

### 2.3. Extracellular Vesicles

The therapeutic effect of stem and progenitor cells is attributable not only to their engraftment capacity or their ability to differentiate at the site of damage but due to the paracrine mechanisms which they exert [34]. It is established that this activity is a result of EVs [23]. 

EVs are membrane-derived particles surrounded by a phospholipid bilayer that are released by cells. In addition to direct cell-to-cell contact and the secretion of soluble factors, EVs also function as a mechanism of intercellular communication. These vesicles are able to efficiently deliver their parental cell-derived molecular cargo to recipient cells—in this case, the respiratory epithelium, which can result in subsequent structural changes at an RNA, protein, or even phenotypic level. EVs are similar in many ways to liposomes in terms of their physical characteristics, in vivo behavior and fate, cellular interactions, and cargo loading, and are thought to hold significant potential in drug delivery applications [35]. 

EVs offer a unique, dynamic communication network for cells owing to their ability to transport molecules such as cytokines, proteins, lipids, miRNAs, mRNAs, chemokines, and ncRNAs, altering the functions of the recipient cell [36,37]. 

There has been great interest in EVs from researchers owing to their potential to be used as drug delivery systems, enhancing the target delivery of drugs and surpassing standard drug delivery methods [38]. The advantages of using EV alternative drug delivery systems have been demonstrated in preclinical models, which have shown a low toxicity; high target efficiency; and the ability to escape degradation, resulting in a slower clearance from the body [39,40]. 

EVs obtained from cells are classified based on their size and source; exosomes are a type of EV, along with micro vesicles and apoptotic bodies, that are 30–100 nm in diameter and are not only secreted by MSCs but also by other stem cells, such as induced pluripotent stem cells (iPSCs), embryonic stem cells (ESC), hematopoietic stem cells (HSC), and neural stem cells (NSC) [39,41]. Though these types of stem cells are considered to be safe to use as therapeutics, there are some biosafety concerns associated with their application, such as immune-rejection, tumorigenicity, and the potential for abnormal differentiation [42]. Exosomes are suggested as an alternative to stem cell application and are paid increasing attention in studies due to their ability to mimic the effects and the phenotypes of parental cells without the associated risk factors [22,43,44]. 

The exosomes obtained from MSCs have the ability to mirror the same functions as the mother cell, promoting the repair and regeneration of tissues and cells and restoration homeostasis [45]. It is reported that MSC-derived exosomes are responsible for the paracrine mechanism of MSCs and deliver a biological effect equal to MSCs themselves [46]. MSC-derived exosomes are suggested to have many advantages in contrast to MSCs in the effect that they have on target cells by acting directly with them; the decreased risk of tumorigenicity and immune rejection; their ability to be maintained in cryostorage for a long period of time due to the presence of the plasma membrane; and the ease at which variable factors can be controlled, such as the concentration, dose, and route of administration [46,47]. These properties indicate their potential safe application for respiratory disease therapies which has already been demonstrated in preclinical models [48,49]. Despite this intriguing potential, EVs are not without their risks, with reported detrimental roles in angiogenesis, immunosuppression, and metastasis [50].

## 3. Respiratory Diseases

Respiratory diseases are among the most common acute and chronic diseases worldwide which occur in both developed and developing societies and are present in among all age groups [51]. Respiratory diseases arise from different factors, such as the inhalation of toxic agents, harmful lifestyle, infection, or genetic factors [51]. Some of the most common disease effecting the respiratory system include acute respiratory distress syndrome (ARDS); chronic obstructive pulmonary disease (COPD); asthma; sepsis; and the more recently described COVID-19, following infection with the Sars-CoV-2 virus.

### 3.1. Acute Respiratory Distress Syndrome

ARDS is a debilitating disease of the lungs which has a high mortality rate [52]. ARDS arises from a variety of different etiologies, but its most commonly recognized and fatal cause is the pathogenic infection known as pneumonia [53]. Pathogens such as Escherichia coli, Streptococcus pneumoniae, Klebsiella pneumoniae, and Staphylococcus aureus lead to the development of pneumonia causing inflammation, the infiltration of leukocytes to the airspace, and the impaired oxygenation of the blood [53,54].

The complex pathophysiology and development of ARDS makes it difficult to develop therapies for treatment. Current interventions include ventilation, fluid management, antibiotics, and neuromuscular blockade [55,56].

### 3.2. Chronic Obstructive Pulmonary Disease (COPD)

COPD is a global healthcare problem that is anticipated to become a growing problem as the population ages and the use of tobacco increases. In 2016 in the US, COPD was the fourth leading cause of death, following heart disease, cancer, and cerebrovascular disease, with a total of 251 million reported cases globally and 3.17 million deaths [57]. The major risk factor associated with COPD is the inhalation of toxic chemicals and gases, which leads to the inflammation of the lungs and airways, causing parenchymal destruction and the destruction of lung tissue known as emphysema [58]. There are currently no medicines available that have demonstrated the ability to inhibit the progressive deterioration associated with the disease. The regular use of inhaled bronchodilators is intended to alleviate symptoms associated with COPD [59].

### 3.3. Asthma

Asthma is a non-communicable chronic inflammatory disease which presents as a lifelong condition and affects all age groups with different severity throughout the course of the asthma patient’s life [60]. According to the Global Asthma Report, in 2018 asthma effected 339 million people worldwide and the prevalence is continuing to rise [61]. The disease is made up of heterogenous phenotypes that present differently in terms of their etiology and pathophysiology, with risk factors including genetics and the environment [60].

Asthma is linked to T help cell type-2 (Th2) immune responses and may be triggered by allergen stimuli factors such as dust mites and pollen, and perennial allergens such as viral infections, cold air, and exercise. These factors can induce a series of events which leads to chronic airway inflammation [62,63]. Elevated levels of Th2 cells in the airways release specific cytokines leading to bronchospasm, an increase in mucus secretion and edema [64]. Current measures to manage asthma only include pharmacological intervention through the use of inhaled corticosteroids (ICS) [65].

### 3.4. Sepsis

Sepsis presents a significant problem associated with high morbidity and mortality rates and is one of the leading causes of deaths amongst critically ill patients in non-coronary intensive care units in the US [66]. There are various aberrations that underpin sepsis which involve different organs and systems; one of the most established is an uncontrolled inflammatory response [67,68]. 

Sepsis occurs due to infection which develops past local tissue and causes a cascade of physiological responses which ultimately lead to organ dysfunction [67]. Respiratory tract infections such as pneumonia are the most common associated infection source, and as respiratory dysfunction develops so do complications such as ARDS [69]. Patients with sepsis experience a loss of hypersensitivity and an inability to clean infection and become predisposed to nosocomial infections [70]. 

Current treatments to improve the condition include early recognition, antibiotics, lung protection ventilation, and methods to reduce nosocomial infections, however, due to the complex nature of sepsis, there is no standardized approach [71]. Despite advancements in the understanding of the pathophysiology of sepsis, there has been no therapeutic agents approved for its treatment and therefore there is an obvious need for the development of novel therapeutic strategies.

### 3.5. COVID-19

Sever acute respiratory syndrome coronavirus 2 (Sars-CoV-2) has been identified as the agent responsible for the disease Coronavirus disease 2019, also known as COVID-19, which has quickly developed into a global pandemic [72,73]. On March 11^th^ 2020, the World Health Organization (WHO) declared the virus a public health emergency two months following the first identified case in Wuhan, China. COVID-19 primarily targets the lungs [74]. Under normal conditions, the lungs are protected by infiltrating inflammatory cytokines such as macrophages, however this defence system can become over-stimulated in response to harmful agents, resulting in an uncontrolled inflammatory response and causing severe damage to lung tissue [75]. The hyper response known as a “cytokine storm” is central to the progression of COVID-19 and is supported by a complex coordinated process. The process involves: (i) the activation of antigen-presenting cells such as macrophages, which alter the appearance of lymphocytes to the virus; (ii) the activation of pro-inflammatory factors caused by viral RNA replication within the host cell; (iii) lymphocyte apoptosis and immune evasion caused by lymphocyte viral invasion [76,77].

At the time of the publication of this review, there is no vaccine or treatment options available for COVID-19, with prevention of infection and supportive care as the current measures in place [78]. Studies are exploring the potential use of antivirals, antibiotics, and agents such as hydroxychloroquine, however these options are single-target agents and, owing to complexity of the disease, a pleiotropic agent is likely required in order to maximize the therapeutic effect across a range of potential therapeutic strategies, which may influence any of antigen-presenting cell activation, viral RNA replication with cells, and lymphocyte apoptosis, for example [79]. The impact COVID-19 has had on both global health and the economy has been immense and will continue to be unless a novel, effective therapeutic is developed. The use of cellular therapies such as MSCs and exosomes holds great promise as an alternative approach for the treatment of COVID-19, owing to the protective and reparative effects for pulmonary damage and the ability to suppress host antiviral defenses which characterizes COVID-19 [75,79,80].

## 4. Routes of Administration for the Treatment of Respiratory Diseases

The efficiency of lung treatment depends on a variety of factors, including the route of administration by which the therapeutic is delivered. Various drug delivery systems have been developed to minimize harmful side effects and to maximize the therapeutic effect of the drug used [81] with inhalation therapy as the most commonly used route of administration for the delivery of drugs for the treatment of respiratory disease [82].

Selecting inhalation as the primary administration route allows the direct targeting of drugs to the airways, permitting the rapid absorption of low-dose medication and potentially lowering the incidence of systemic bioavailability [83]. In contrast, intravenous (IV) administration involves the direct injection of drugs to the blood vessels; by doing this, the amount of available drug is affected by gastrointestinal absorption and hepatic first pass effects before it reaches the lung [84]. Additionally, it is thought that there is aggregation or clumping of EVs and MSCs in the injured lung microvasculature, and this carries the risk of mutagenicity and oncogenicity [85].

Inhalation therapy allows a higher local delivery drug concentration at lower doses to the lungs than that achieved by IV administration, potentially making this non-invasive delivery method more economical due to the requirement for a lower dosage of expensive drugs [82].

Overall, the high pulmonary efficacy, rapid onset, and reduced risk of adverse effects achieved by inhalation therapy is superior to that achieved by delivery to systemic circulation through IV or oral administration [86].

The direct targeting of the lungs and rapid onset is achieved through the use of aerosol generating devices, which include, but are not limited to, pressurized metered dose inhalers (pMDI); dry powder inhalers (DPI); soft mist inhalers (SMI); and nebulizers, which are either air-driven jet nebulizers (JN) or the newer closed-circuit vibrating mesh nebulizers (VMN).

### 4.1. Combination Product Development—Selecting the Delivery Device

Any combination product development is considered a complex undertaking, but a prudent approach to the development of ATMP/device combinations should begin with the screening and selection of the aerosol generator devices from early in the program. The following must be considered as a whole, as it will have a critical bearing on the ultimate choice of device. Some devices will not be suitable for use with the patient or patient intervention, or even the therapeutic itself.

Patient type: the patient may be any of adult, pediatric, or infant. In combination with the disease state, the patient type will have a direct bearing on the breathing parameters (breath per minute, tidal volumes, inhalation to exhalation ratio, peak inspiratory flow rate), which in turn can affect the aerosol dose delivered to the lung [87,88]. The infant lung is considered the most difficult to administer appreciable amounts of therapeutics to, and treatment options may be very limited [89]. 

Patient interventions during which the ATMP will be administered: these include oxygen supplementation via a mouthpiece or face mask in spontaneously breathing patients, or ventilatory support either through invasive or non-invasive means (full masks, nasal pillows, nasal cannula). Each intervention will have a direct bearing on the lung dose, and in some instances the device may not be suitable for concurrent usage. For example, DPI is not suitable for use during mechanical ventilation or high-flow nasal therapy. Another consideration may be for how long the patient is in receipt of the ATMP therapy. In this instance, continuity of technology is important. If therapy begins prophylactically, and continues through the escalation of the disease, a variety of patient interventions may be encountered. A review of the literature would suggest that nebulizers are the only technology that can be used concurrently across all of these interventions; however, as will discussed below, VMN consistently delivers the largest amount of formulation to the lung across the interventions, and thus presents an example of how the appropriate selection of delivery device/technology can streamline combination product development and potentially facilitate expanded ATMP prescription throughout the disease course.

Targeted location within the respiratory tract: depending on the intended site of action, the aerosol droplet size will be a key determinant in getting the therapeutic to where it will have greatest impact. Generally, lower droplet sizes penetrate deeper and towards the lung periphery than larger droplet sizes do. In the case of simple bronchodilators, appropriate targeting through the selection of aerosol droplet sizes has been shown to significantly enhance inhaled drug therapy [90]. Patient-related factors such as breath pattern, position, and disease condition are also known to influence aerosol deposition and distribution [88,91,92,93].

Formulation design: generally, formulations for inhalation are liquid solutions, liquid suspensions or dry powders. Relating to ATMPs, their efficacy will likely be determined by the number of cells or exosomes, as opposed to a mass of therapeutic acting on a milligram per kilogram basis. As such, it is highly unlikely, for example, that stem cells would be administered as a dry powder (via DPI) or in the low, microliter range dose volumes characteristic of pMDI and SMI. That said, spray drying has been shown to be feasible in the case of exosomes, and, if sufficient quantities could be delivered, could be suitable for MSC-derived proteins and peptides [93].This notwithstanding, nebulizers are likely the most appropriate choice for use in the critical care setting. However, within the nebulizer choices, there are potential technological limitations—e.g., JN preferentially aerosolize the suspension buffer. Stem cells and even exosomes are suspensions, and therefore potentially unsuitable for bespoke commercial combination with a JN for the economic reasons of wastage. However, the use of JN has been reported to be compatible with MSCs [94]. Table 1 summarizes the formulation characteristics of ATMPs and their potential compatibility with the different aerosol generators alone. As mentioned, however, compatibility alone does not necessarily mean that a device is fit for purpose—for example, the patient intervention needs to be considered in the final device selection for the combination product.

Delivered lung dose and ATMP cost: ATMPs are expensive to produce, and the cost of therapy will be high, with currently approved, non-respiratory ATMPs ranging from tens of thousands to more than a million dollars per treatment [102]. Technological limitations again will limit the adoption of JN, considering that they have a relatively poor performance, with residual doses, not available to the patient, ranging from 1.0 to 1.8 mL. Depending on the nominal dose originally placed in the JN, this may equate to as little as 40% escaping as potentially inhalable aerosol, with 60% of the dose, by volume, retained in the nebulizer never to become available to the patient [103,104]. In this instance, if a JN is chosen as the delivery device, there is the potential for more than half of the therapy to never leave the nebulizer, thus effectively doubling the cost of a dose or treatment. VMN, on the other hand, has emitted doses in excess of 95% of the nominal dose, and so immediately minimizes wastage [105].

The dose emitted by a device is taken into the breath and inhaled into the lung. The total dose deposited in the lung has been shown to vary massively with device selection. Using JN and VMN as examples again, multiple-fold differences can be seen on the amount of therapy delivered to the lung, which in turn impacts the potential for therapeutic benefit. One Single-photon emission computed tomography (SPECT-CT) comparison of lung deposition in spontaneously breathing adults demonstrated this clearly, with 5.2 ± 1.1% of the nominal dose initially placed in the nebulizer being delivered with a JN versus 34.1 ± 6.0% being delivered with a VMN in the same patient population (a 6.56-fold difference) [103].

One commercial consideration should also be ability to supply the technology, or secure supply at the levels required should the ATMP be a success. Simply naming a general purpose, off the shelf device on a label is to be considered a risky strategy on the basis that, in the future, that device may for whatever reason become unavailable. A co-developed combination product will be developed for the specific patient population and disease target in mind. This approach reduces risk on the basis of a known “built in” reliable and reproducible performance, as well as agreed security of the supply of both the ATMP and device. Additionally, another commercial consideration would be whether the manufacture of the device can scale at the required levels, or whether there is sufficient intellectual property coverage for that device.

Finally, caregiver and by-stander safety must be considered. The recent renewed focus on fugitive emissions during respiratory therapy has highlighted the need for several other potential product requirements. Mitigating, preventing, or capturing exhaled medical or therapeutic aerosols or even patient-derived bioaerosol has become a critical requirement. Several recent studies highlight the risks, the differences between devices, and the steps that can be taken to reduce the risk of unintended inhalation of fugitive emissions. Briefly, the guidance is that closed-circuit nebulizer systems, such as vibrating mesh nebulizers, should be used, and open circuit nebulizer systems, such as jet nebulizers that require a break in the patient circuit should be avoided [106,107,108,109,110].

### 4.2. The Feasibility of Aerosol-Mediated ATMP Delivery

As discussed, MSCs have offered great potential for the treatment of respiratory disease, and their safety has been demonstrated in both preclinical and clinical trials. However there are some problems associated with the administration of the cells, such as the potential for the delivery method to adversely affect the therapeutic effect of the cell, or the failure of the cell to home directly to the target organ, leading to off-target effects [111,112]. Of note, during our review of the literature, there was yet no record of the use of aerosol-mediated delivery to the lung for systemic or non-lung targeting. The aerosolization of MSCs and their products has been shown to overcome these associated issues [113]. One study using a VMN demonstrated the successful delivery of MSC-derived CM for the treatment of ARDS while maintaining its antibacterial properties [100]. Moreover, other studies have shown the ability of MSCs to maintain their engraftment potential following nebulization [99]. Table 2 lists some of the recent COVID-19 clinical trials that use aerosol-mediated delivery. A recent state of the art review of the application of ATMPs in respiratory disease and the use of aerosol delivery provides a comprehensive listing of active ATMP trials in respiratory disease [114].

## 5. Rationale for Aerosol-Mediated Delivery of Cellular Therapies

Owing to the complexity of the respiratory system, following exposure to different damaging elements such as toxic gases, microparticles, and carbon granules, the system can become severely damaged to the point where it may not return to its original state [115]. There has been a significant increase in the prevalence of respiratory diseases such as COPD and asthma, and yet there are no curative treatments available for these conditions [116,117].

As previously discussed, these respiratory diseases occur due to chronic inflammation and emphysema [58,67]. Cellular therapies offer a novel approach for the treatment of these diseases owing to their anti-inflammatory, anti-fibrotic properties and their ability to repair and replace damaged structures within the lung, such as bronchioles and alveoli, as seen in patients with emphysematous [84,118]. In recent years, the focus of research has been directed towards MSC-based products due to their diverse mechanisms of action [118,119].

In respiratory diseases such as COPD and ARDS in which inflammation plays a fundamental role, the paracrine mechanism, which MSCs exert, modulates the proliferation and activation of immune cells, which themselves are central players in the pathogenesis of both chronic and acute inflammatory lung disease [21,120]. In respiratory disease where lung tissue becomes damaged, MSCs also have the ability to assist lung regeneration by secreting cytokines and growth factors to replace damaged alveolar epithelial cells and restore endothelial permeability [121,122]. Moreover, cellular products such as EVs and CM are under investigation owing to their small size, which means they can be delivered for the treatment of respiratory diseases by non-invasive methods such as inhalation [23].

Importantly, there have been no reported adverse side effects associated with MSC therapy; however, it has been noted that, in parallel with their therapeutic potential, malignant transformation remains a potential risk. The risk is considered highest with embryonic stem cells and induced pluripotent stem cells [123]. As outlined above, aerosol-mediated delivery is possible at all stages throughout the development of the disease, from spontaneous breathing patients (via mouthpiece and facemask), to those in receipt of supplemental oxygen—e.g., via high-flow nasal therapy (via nasal cannula, nose mask) and those patients requiring ventilatory support by either non-invasive or life-sustaining invasive mechanical ventilation (via facemask, hood, tracheostomy tube, endotracheal tube) [124]. The topical administration of aerosolized ATMPs allows for high local concentrations directly at the site of action, as well as the avoidance of the well-documented disadvantages of systemic, gastrointestinal administration. Interestingly, beyond hepatic first losses being a major blockade, for ATMPs it has been proposed that aerosol-mediated delivery helps overcome the risks associated with aggregation in the microvasculature. Considering these factors in combination, aerosol-mediated delivery has the potential to profoundly impact the future of cellular therapies in a clinical setting [23,125,126].

## 6. Regulatory Framework for ATMP/Device Combination

As with any marketed medicinal product, certain controls during the design and manufacture need to be applied and complied with. Drug device combination products are commonplace, and certainly within the respiratory drug delivery field—for example, dry powder inhalers are specific, co-developed combinations of both a specific drug and a specific device. The same level of development effort and control should be applied to ATMP device combinations in an effort to ensure maximal efficacy and safety.

Within the EU and America, there are an outlined set of legal guidelines in place which oversee the regulation of medicinal products intended for human use, including ATMPs [127,128]. The regulatory framework in place is intended to guarantee the safety, efficacy, and quality of all medicinal products entering the market of each region [128]. The translation of an ATMP from research to clinical trials to product approval is a prolonged process due to not only both scientific and practical obstacles but also regulatory challenges [129]. The development of an ATMP begins with its classification early in the process; this will govern the regulatory guidelines and recommendations which must be followed for approval [127].

### 6.1. EU Requirements

Regulation plays a crucial part in the translation of novel medicinal products and medical devices from R&D to clinical application, ensuring that all products meet a high quality standard [127]. Within the EU, there is a wide range of legal frameworks in place for medicinal products for human use, which range from products containing chemical substances or biological substances, which include immunological products; products of human origin, such as human plasma; and biotechnological products, such as ATMPs [127].

In the EU, Directive 2001/83/EC and Regulation 726/2004/EC were the regulatory framework structures in place to define a medicinal product intended for human use [128]. These guidelines were revised and, in 2008, new amended regulatory guidelines under Regulation 1394/2007/EC were established specifically for the regulation of ATMPs [3]. The established ATMP regulation outlines regulatory principles for the evaluation, authorization, and post authorization of ATMPs intended for human use so that all member states of the EU follow the standards consistently [112,114]. Regulatory bodies who oversee the regulation of ATMPs include the Committee for Advanced Therapies (CAT) established by the European Medicines Agency (EMA). CAT is a specialized committee responsible for all ATMP-associated activities such as the evaluation of ATMP marketing authorization (MA), assisting scientific developments, as well as offering scientific advice on the classification of ATMPs [128,130].

Before a medicinal product enters the market, an MA is required by competent authorities to guarantee that medicinal products are safe for human use [128]. The Market Authorization Application (MAA) procedure evaluates the quality, safety, and efficacy of all data gathered throughout clinical development, from the proof of concept stage to the human clinical trial stages, ensuring regulatory standards are met [128]. In order to obtain MA approval, the manufacture must comply with the standards set out in both Directive 2001/83/EC and Regulation 726/2004/EC, and the medicinal product must be manufactured in accordance with Good Manufacturing Practices (GMP) guidelines [131].

The end point of the evaluation of data gathered from clinical development is to establish the benefit to risk ratio of the medicinal product; authorization can then only be granted if a positive result is obtained [132]. Due to the human cell-based nature of ATMPs, Directive 2009/120/EC outlines the supplementary clinical and non-clinical data required during the developmental stage [5].

Once safety and efficacy standards are met, in order for ATMPs to reach the market for commercialization a central MA must be secured under the centralized procedure by submitting the MAA to the EMA, which results in product authorization in all EU states and European Economic Area (EEA) regions [128]. There are three different potential routes to be taken for MA, the standard, conditional MA and MA under exceptional circumstances. A standard MA is the most commonly granted MA permitted once a positive benefit risk ratio is demonstrated from generated clinical data [128].

Regarding the regulation of medical devices within the EU, Directive 93/42/EEC on medical devices (MDD) was one of three established key legal frameworks in place that outlined the regulatory standards for the authorization of medical devices [133]. However, the MDD has since been replaced with the Medical Device Regulation (MDR) 2017/745 that requires more in-depth conformity assessments and Clinical Evaluation Reports (CERs), creating a challenging Conformitè Europëenne (CE) marking process, albeit resulting in the safe and effective medical device released onto the EU market [134]. Of note, due to the COVID-19 pandemic, the MDR will not become fully implemented until 26 May 2021.

Similar to ATMPs, the regulation of medical devices begins with classification early within the development phase [135]. The classification of medical devices is outlined in Annex IX of Directive 93/42/EEC; following these guidelines, the manufacturer must categorizes a device into one or four classes relative to the device characteristics and intended application: Class I, IIa, IIb, and III; see Table 3 [136]. Nebulizers are the only general purpose, non-paired device on the list of aerosol generators. pMDI, DPI, and SMI are themselves part of a drug–device combination. General-purpose nebulizers are considered Class II.

Before entering the market, medical devices must be Conformitè Europëenne (CE)-marked to demonstrate that the device meets the regulatory standards outlined in the MDR and any other relevant EU legislation. Once the device is classified and meets the regulation requirements set forth, the manufacturer must submit an application to a notified body to prove that the device meets the requirements for CE markings [134]. A NB is an organization within the EU appointed to evaluate the conformity of products prior to entering the market [137].

The NB will request certain documentation depending on the device classification and will assess whether the conformity is in compliance with the requirements outlined in the MDR [138]. The manufacturer may be required to implement a quality management system (QMS) in accordance with the MDR (Annex VIII), which includes clinical evaluation, Post Market Surveillance (PMS), and Post Market Clinical Follow-up (PMCF) plans [139]. Moreover, the manufacturer is required to provide CE technical documents that support conformance claims [140]. Following approval, post-market surveillance will be carried out by the relative competent authority to ensure that the safety and effectiveness of medical devices are maintained [138].

### 6.2. US Requirements

Within the US, the Food and Drug Administration (FDA) is the regulatory body responsible for the approval and authorization of medicines entering the market. The federal food, drug, and cosmetic act (FDCA) and the Public Health Service Act (PHSA) are the two primary statutes which legally authorize the FDA with the responsibility of the regulation of human medicinal products such as biological devices and medical devices [127,141].

Similar to the EU, in the US the regulation of ATMPs follows the same framework as biological products, including allergenics, blood products, vaccines, and cell and gene therapy products; the regulatory standards are outlined in Section 351 of the PHSA and under the FDCA [141]. The FDA standards are outlined within the Code of Federal Regulation (CFR), where the regulatory standards for biological and medical devices are outlined under Title 21 of the CFR [141]. There are nine FDA organization centers responsible for product jurisdiction, with the Center for Biologics Evaluation and Research (CBRE) having jurisdiction over biological products, including ATMPs, as well as devices and combination products [141,142].

The 21^st^ Century Cures Act implemented in 2016 was established to assist in the development of ATMPs and saw the establishment of the Regenerative Medicine Advanced Therapy (RMAT) designation developed to assist cellular therapies, tissue-engineered products, and combination products which use biological products as the operating principle [10,143]. According to the act, a drug qualifies for RMAT designation if (I) the drug is a regenerative medicine; (II) the drug’s intended use is for the treatment, modification, reversal or cure of a life-threatening disease or condition; or (III) the drug shows potential to treat diseases or conditions with unmet medical needs based on clinical data [128].

For all medicinal products, including drugs, biologics, and devices, the FDA-outlined good practices (GxPs) must be followed throughout both the manufacturing stage and the release stage, ensuring the safe and effective release of high-quality products. The GxPs include Good Manufacturing Practices (GMP)/Quality System Regulation (QSR), Good Laboratory Practice (GLP), Good Clinical Practice (GCP), and Good Tissue Practice (GTP). The principle of GMP is a basic regulatory requirement to be followed during the manufacturing of drugs and biologics, while QSR would apply to medical devices or the device constituent of a biologic/device combination product [141].

Outlined in Section 3034 of the act are the standards to be followed by the FDA in the evaluation of devices which operate in the recovery, isolation, or delivery of regenerative medicine advanced therapies. The regulatory pathway for devices varies subject to the technical qualities and the intended application of individual devices [144]. The class which the device is categorized into is the primary factor which determines the appropriate pathway to choose for market approval. A device can be classified into one of three groups based on the level of regulation required to assure appropriate safety and effectiveness. The three category groups include: Class I, Class II, and Class III (Table 4) [144].

Following the classification of the device, there are four premarket pathways available that depend on different characteristics associated with the device: (i) de novo classification request, (ii) premarket approval pathway (PMA), (iii) Humanitarian Device Exemption (HDE), (iv) premarket notification (501 (k)). The latter is the appropriate pathway chosen for a device where a device of the same type has formerly been granted marketing approval and has been recognized as both safe and effective by the FDA [144].

The premarket notification (510 (k)) pathway is chosen when a device is being introduced onto the market for the first time, or when there are modifications made to an existing marketed device which may impact both the safety and capability of the device [144]. Any changes made to the device must be done in compliance with the quality system regulation (21 CFR 820) and documented [145]. The device to be approved must be compared to an alike existing marketed device known as a predicate in order to prove that it is substantially equivalent (SE). In order for the device to be classified as SE, it must demonstrate the same technological features and specified application as the predicate [144].

In the case of a nebulizer, devices which meet the definition of a nebulizer are outlined in 21 CFR 868. A total of 5630 of which are capable of using legal market drug formulations in a way which is compatible with a labelled formulation and must be evaluated in accordance with device law and regulation [146,147]. For a nebulizer to support substantial equivalence claims, the device must be characterized in vitro and compared to the in vitro data of the predicate device under the Good Laboratory Practice standards [145]. Comparison of the released aerosol from the mouthpiece is a performance characteristic which must be evaluated and includes the total emitted mass, dose delivery uniformity, and particle size distribution [147,148].

However, when a nebulizer is considered a part of a new drug/device combination for the delivery of a new formulation such as cellular products, this requires evaluation by the Center for Drug Evaluation and Research (CDER), as the primary mode of action (PMOA) is the medicinal product. In this case, device performance is evaluated by specialist subcommittees which may be provided by the likes of the Centre for Devices and Radiological Health (CDRH). These performance factors to be evaluated include performance characterization in in vitro and in vivo studies, device safety and efficacy, and clinical study data [131].

## 7. Conclusions

The aerosol-mediated delivery of ATMPs for the treatment of respiratory disease is under investigation in pre-clinical and clinical studies, and the results obtained from on-going clinical trials will have a significant impact on the future treatment of acute and chronic respiratory diseases. As ATMP/device combination products consist of separate components that are regulated by different health authority divisions, there are many scientific and regulatory challenges which must be overcome before widespread commercial availability. It is vital for manufacturers and regulatory bodies to collaborate and maintain transparency during product development to ensure that products entering the market are at a highly safe and effective standard.

The regulatory roadmap detailed above is intended to serve as a summary guidance to researchers and developers in an effort to streamline the development process. Early-stage researchers can look to de-risk development programs through the early selection of appropriate delivery technologies.

## Figures and Tables

**Table 1:** Summary of Advanced Therapeutic Medicinal Product (ATMP) formulation characteristics and their compatibility with aerosol generators.

ATMP and Its Formulation Characteristics					
		Stem Cell	Secretome	Exosome	
		Micro suspension of cells	Mixture of suspended exosomes and solubilized proteins and peptides	Nano suspension of exosomes	
**Aerosol Generator**	**Typical Dose**	**Potential ATMP Compatibility with** **Aerosol Generator**			**Supporting References**
Dry Powder Inhaler (DPI)	8–40 mg	No	Yes	Yes	[95,96]
Pressurized Metered Dose Inhaler (pMDI)	25 to 100 μL	No	Yes	Yes	[85]
Soft Mist Inhaler	15 μL	Yes	Yes	Yes	[97]
Jet Nebulizer (JN)	2 to 6 mL	Yes	Yes	Yes	[94,98]
Vibrating Mesh Nebulizer (VMN)	0.1 to 50 mL	Yes	Yes	Yes	[99,100,101]

**Table 2:** Clinical trials using inhalation as the delivery method for COVID-19 as an exemplar respiratory disease.

NCT No.	Title	Enrolled	Intervention	Dose	Phase	Status
NCT04276987	A Pilot Clinical Study on Aerosol Inhalation of the Exosomes Derived From Allogenic Adipose Mesenchymal Stem Cells in the Treatment of Severe Patients With Novel Coronavirus Pneumonia	30	Single-group assignment	MSC derived exosomes 5 Times aerosol inhalations of MSCs-derived exosomes (2.0 × 10^8^ nano vesicles/3 mL at Days 1, 2, 3, 4 and 5	Phase 1	Not yet recruiting
NCT04389385	Aerosol Inhalation of the Exosomes Derived From Allogenic COVID-19 T Cell in the Treatment of Early Stage Novel Coronavirus Pneumonia	60	Single-group assignment	Specific T cell-derived exosomes (CSTC-Exo) Aerosol inhalation of CSTC-Exo (2.0 × 10^8^ nanovesicles/3 mL at Day 1,2,3,4 and 5, 5 times daily	Phase 1	Active, not recruiting
NCT04473170	Study Evaluating the Safety and Efficacy of Autologous Non-Hematopoietic Peripheral Blood Stem Cells in COVID-19 (SENTAD-COVID)	146	Parallel assessment	Autologous Non-Hematopoietic Peripheral Blood Stem Cells (NHPBSC) therapy as add-on COVID-19 standard care	Phase 1 Phase 2	Completed

**Table 3:** Product classification and routes for the authorization of medical devices within the EU [135].

Class	Risk	Description	Route
I	Low risk	Non-invasive, does not interact with body	Self-declaration route outlines in Annex VII Module. An EU Declaration of Conformity—manufacturer must declare the device satisfies the provisions of the directive.
IIa	Medium	Generally invasive Therapeutic transfer of energy to patient or used for the diagnosis or monitoring of medical condition	Carry out conformity assessment carried out by NB using Annex II, IV, V, or VI. Can declare conformity with the provisions of directives and regulations (Annex VII).
IIb	Medium	Invasive—surgical or implantation Potential to modify body fluid	Full quality assurance (Annex II)—with assessment by NB of technical documents in one sample in accordance with the directive (Annex II, Section 7).
III	High	Invasive—used to sustain human life, essential to maintain human health Devices connected to CNS or CCS	Full quality assurance system audit (Annex II)—full examination of device by a Notified Body (NB)

**Table 4:** The three groups in which devices can be classified into under Food and Drug Administration (FDA) rules [144].

Class	Description
I	Devices are subject to a comprehensive set of regulatory authorities called general controls that are applicable to all classes of devices.
II	Devices for which general controls, by themselves, are insufficient to provide reasonable assurance of the safety and effectiveness of the device, and for which there is sufficient information to establish special controls to provide such assurance.
III	Devices for which general controls, by themselves, are insufficient and for which there is insufficient information to establish special controls to provide reasonable assurance of the safety and effectiveness of the device. Class III devices typically require premarket approval.
